# Surface Microstructures on Planar Substrates and Textile Fibers Guide Neurite Outgrowth: A Scaffold Solution to Push Limits of Critical Nerve Defect Regeneration?

**DOI:** 10.1371/journal.pone.0050714

**Published:** 2012-12-12

**Authors:** Stefan Weigel, Thomas Osterwalder, Ursina Tobler, Li Yao, Manuel Wiesli, Thomas Lehnert, Abhay Pandit, Arie Bruinink

**Affiliations:** 1 MaTisMed, Materials-Biology Interactions Lab, EMPA Materials Science and Technology, St. Gallen, Switzerland; 2 Technische Universität München, Zoology, Freising-Weihenstephan, Germany; 3 National Center for Biomedical Engineering Science, National University of Ireland, Galway, Ireland; 4 Department of Biological Sciences, Wichita State University, Wichita, United States of America; 5 Ecole Polytechnique Fédérale de Lausanne (EPFL), Laboratory for Microsystems 2, Lausanne, Switzerland; Rutgers University, United States of America

## Abstract

The treatment of critical size peripheral nerve defects represents one of the most serious problems in neurosurgery. If the gap size exceeds a certain limit, healing can't be achieved. Connection mismatching may further reduce the clinical success. The present study investigates how far specific surface structures support neurite outgrowth and by that may represent one possibility to push distance limits that can be bridged. For this purpose, growth cone displacement of fluorescent embryonic chicken spinal cord neurons was monitored using time-lapse video. In a first series of experiments, parallel patterns of polyimide ridges of different geometry were created on planar silicon oxide surfaces. These channel-like structures were evaluated with and without amorphous hydrogenated carbon (a-C:H) coating. In a next step, structured and unstructured textile fibers were investigated. All planar surface materials (polyimide, silicon oxide and a-C:H) proved to be biocompatible, i.e. had no adverse effect on nerve cultures and supported neurite outgrowth. Mean growth cone migration velocity measured on 5 minute base was marginally affected by surface structuring. However, surface structure variability, i.e. ridge height, width and inter-ridge spacing, significantly enhanced the resulting net velocity by guiding the growth cone movement. Ridge height and inter-ridge distance affected the frequency of neurites crossing over ridges. Of the evaluated dimensions ridge height, width, and inter-ridge distance of respectively 3, 10, and 10 µm maximally supported net axon growth. Comparable artificial grooves, fabricated onto the surface of PET fibers by using an excimer laser, showed similar positive effects. Our data may help to further optimize surface characteristics of artificial nerve conduits and bioelectronic interfaces.

## Introduction

Due to nerve injury as result of accidents or tissue resections nerve defects can be obtained which exceed a critical size that spontaneously can be healed. Furthermore, connection mismatches may strongly diminish the regeneration efficiency and life quality improvement. Therefore, the control and guidance of neurite outgrowth on material surfaces have become central topics in biomedically oriented material science. Such parameters strongly dictate the design of nerve regeneration supports such as artificial nerve conduits and of bioelectronic interfaces.

In this respect, neurite growth cones can be steered using intrinsic and extrinsic environmental factors. Diffusible [1] and surface bound molecule gradients [2], [3], [4], chemical surface patterning [5], [6] or mechanical structures like pillars [7], micromachined steps, ridges or groove gratings at the (sub-)micrometer scale are promising as guidance cues [8], [9], [10], [11], [12], [13], [14], [15].

The reaction of nerve cells to topographical guidance cues depends on the shape and dimensions of these cues. For instance, neurite outgrowth was not affected by the presence of up to 400 nm deep grooves of 130 nm width and inter-groove spacing [16]. In contrast, a single protein barrier of 250 nm height represented an adequate barrier to constrict neurite outgrowth [17]. Shallow grooves similar to those of Clark *et al.* [16] but with a width of 1 µm influenced neurite outgrowth [18]. Also parallel aligned electrospun fibers are known to direct neurite outgrowth [19], [20], [21], [22], [23]. We assumed that a minimal certain inter-ridge spacing (or groove width) must be present to not only force the neurites to grow in a certain direction but to specifically steer neurite outgrowth to a predefined target.

We aimed to prove that a topographical design of biomaterials in the micrometer range can be defined to optimally promote neurite outgrowth in a predefined direction. For this, we evaluated the effects induced by ridges of 1.3 and 3.0 µm heights and by varying width and inter-ridge spacing in the range of 5 to 100 µm. Promising groove parameters were thereafter evaluated on textile fibers. We used time-lapse images made at 5 min intervals to trace fluorescent chick embryonic neurite growth. Time-lapse imaging allows analyses of growth dynamics, including the speed of outgrowth and retraction, as well as periods of non-growth. We could prove that micrometer channel-like structures sized at the 3–10 µm range on planar substrates, as well as on fibers, are able to optimally direct and improve net neurite outgrowth.

## Materials and Methods

### Substrate preparation

#### Planar substrates

**Figure 1:**
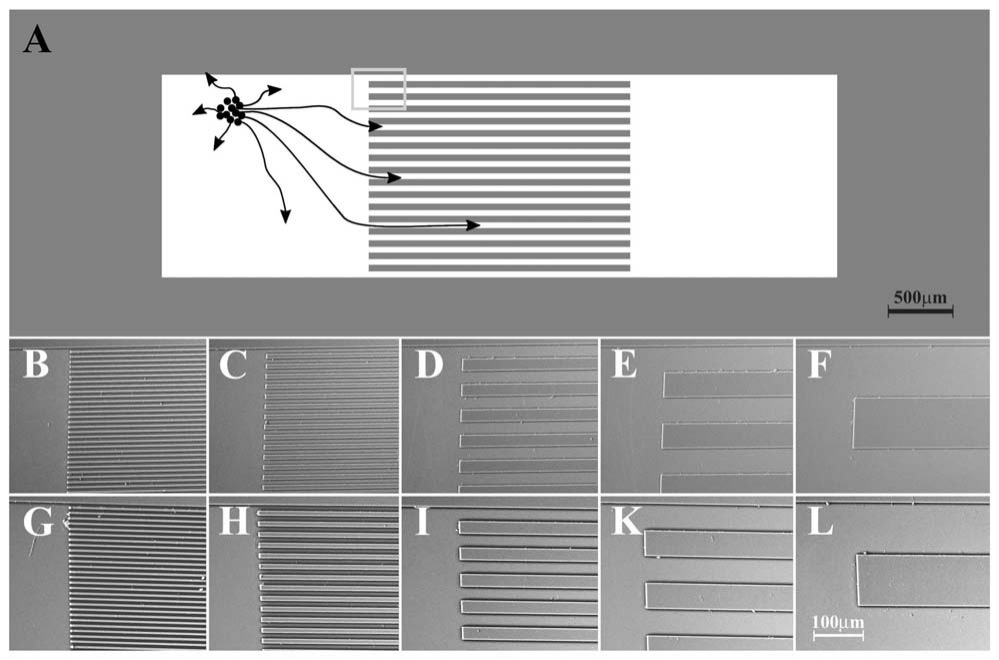
Topographically structured surfaces. A: Schematic representation of a surface consisting of two planar areas and an area with parallel ridges. Growth cones (black arrows) growing out from a reaggregate of spinal cord neurons (black circles, placed in one of the planar areas) into the topographically structured area. The grey rectangle corresponds to the area shown in the images below; the black rectangle is the selected field for live monitoring as shown in Fig. 2. B–L: SEM images of topographical structures, B 5-5; C 10-10; D 25-25; E 50-50 and F 100-100 (ridge width and inter-ridge distance in µm). Structure height: B–F, 1.3 µm.

Oxidized silicon wafers (Si/SiO_2_) were spin-coated with polyimide films (PI). The film thickness was controlled by adjusting the spin rotation speed. Standard processing parameters (PI2610, HD Microsystems, USA) were applied including final curing in an oven at 300 to 350°C for 30 min to complete the imidisation process. Subsequently, the PI films were sputter-coated with a 200 nm thick aluminium layer (Al) which was patterned by photolithographic techniques to create a custom-made etch mask with the desired layout. This pattern was then transferred into the PI films by oxygen plasma etching for about 2 min. Finally, the Al mask was completely removed by an appropriate dry etch step. As shown in Fig. 1, the resulting PI structures consist of parallel ridges with equal ridge widths and inter-ridge spaces of 5, 10, 25, 50 and 100 µm (termed 5-5, 10-10, 25-25, 50-50 and 100-100 in the following). These ridges with heights of 1.3±0.2 µm and 3.0±0.2 µm form channel-like structures on the SiO_2_ surface (termed SiO_2_-PI,). The channels connect two reservoirs, i.e. polyimide-free planar SiO_2_ parts with an area of 1.5×1.5 mm^2^. Ten different channel arrays with the above-mentioned dimensions were fabricated on the same wafer. Individual chips were obtained by dicing the wafer after complete processing. Non-structured PI and SiO_2_ surface areas were used as reference and to investigate the effect of surface chemistry on neurite behavior. Part of the SiO_2_-PI samples were coated with a 40 nm thick layer of amorphous hydrogenated carbon (a-C:H) as previously described [24]. Upon use for cell culture, all samples were coated with 50 µg/mL poly-D-lysine (Sigma Aldrich, CH) in 130 mM Na Borat solution (pH 8.4) for 18 hours at room temperature. Samples were subsequently washed with phosphate buffered saline (PBS; 176.8 mM NaCl, 2.7 mM KCl, 1.47 mM KH_2_PO_4_, 8.1 mM Na_2_HPO_4_, all from Fluka) and coated with laminin (75 µg/ml in PBS, Sigma) for 2 h at 37°C. Samples were used for cell culture experiments within 4 h of the coating procedure.

#### Textile fibers

**Figure 2:**
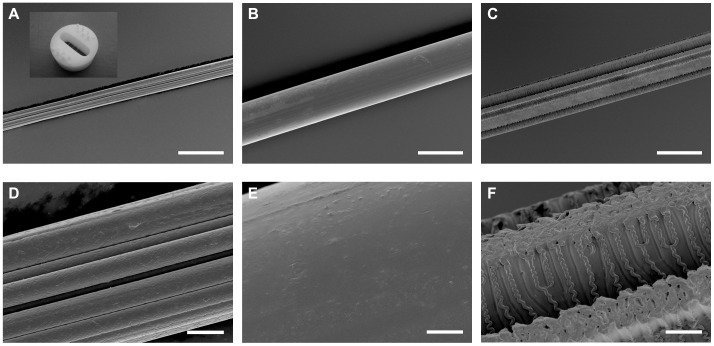
SEM pictures of three particular fibers. Viscose (A, D), PET50 (B, E) and PET50/7 with laser induced grooves of 7 µm depth (C, F); scale bar in A, B & C = 50 µm and in D, E, F = 5 µm. Inset in A shows the fiber holder. Fibers are spanned over the hole in the middle of the device and fixed on the surface by Teflon plugs. Reaggregates are placed nearby the fibers on the coated Teflon planar surface.

Textile fibers were chosen according to material, diameter, surface structure and applicability in experiments (e.g. glass fibers could not be used due to low ductility). Fibers made of polyethylenterephthalat (PET), polylactid (PLA), the two polyamides (PA 6 and 6.6), triacetate (cellulose acetate, CA) and viscose (cellulose, CV) with diameters between 16–21 µm were chosen (for examples see Fig. 2). While the first four fiber types exhibit nearly smooth surfaces, the latter two show longitudinal structures of different dimensions (Fig. 2 A & D). For evaluating effects caused by diameter and resultant curvature of the surface, PET fibers with a diameter of 50 µm were used in addition to the smaller ones. For fixing the fibers and to assure that they do not cross each other, single fibers were fixed onto a Teflon chamber by clamping them into holes using small Teflon pins. The distance between the fibers was around 1 mm. Planar areas on the Teflon holder were chosen as reference.

In a second step, it was investigated how artificially introduced grooves affect (Fig. 2 C & F) neurite growth. PET fibers (50 µm diameter) were structured using an ATL Atlex 193 nm excimer laser in conjunction with an Optec Micromaster machining center. The grooves were 10 µm wide and 3 cm long. For a depth of 2–3 µm, a pulse repetition frequency of 100 Hz, fluence of approximately 0.5 J/cm^2^ and a speed of 3 mm/s was selected to produce each groove. For groove depths of 7 µm, the pulse repetition frequency was increased to 200 Hz, fluence was kept constant at 0.5 J/cm^2^ and speed was 1 mm/s. Each fiber was rotated to form six equidistant grooves. A stainless steel mask with a 0.1 mm×4 mm aperture and a 10-fold optical demagnification was used to produce the channels. Introducing artificial grooves by an excimer laser resulted in grooves with an interior structure. Wagner and Hoffmann [25] observed these ripple structures in stretched PET. They noted that the orientation of the structure was dictated by the orientation of stress in the material and not by the direction of the channel which was perpendicular to the stretching direction of the fiber. They stated also that amplitude and period of structures depend on fluence as well as on number of shots. Therefore the repetition rate, fluence and speed were varied on our samples. However, ripple structures were present for both chosen parameter sets.

Before starting the cell culture experiments, the Teflon chamber with the clamped fibers was incubated twice in 70% ethanol for at least 30 minutes with a vacuum plasma activation step in between (2 minutes at 2–3 mbar and high RF level, PDC 32G, Harrick Scientific Corp.). They were coated with poly-D-lysine and laminin as described above.

### Neuronal cell culture

Chicken eggs were incubated at 37°C for 70 h and, subsequently, spinal cord neurons of chicken embryos were transfected *in ovo* with a modified RFP-plasmid vector pRFP-N1 (Clonetech, USA) as previously described [26]. Briefly, the plasmid was injected *in ovo* into the spinal cord channel followed by an electroporation step (5 pulses with 25 V each for 50 ms, BTX Electro Square Porator T820, Axon Lab AG) between stage HH 14 and 17 [27] while the embryo remained in the egg under incubation conditions (37°C).

We also tested transfection of neurons after isolation. Here, 2×10^6^ dissociated neurons were transfected using the Amaxa Nucleofector II with the Chicken Neuron Kit (Amaxa Biosystems). A suspension of 2×10^6^ cells was centrifuged and the pellet resuspended in 100 µl transfection solution mixed with 10 µg plasmid. After transfection, the cell suspension was used for cell aggregate preparation as described later. Since we didn't see any significant effect on neuronal behavior, we pooled the data of both transfection methods.

The plasmid is used for ubiquitous expression of the red fluorescence protein DsRed in order to visualize cells on the opaque surfaces. The variant used contained 32 unknown point mutations in the coding region of DsRed with the purpose of minimizing the intracellular aggregation of the protein products. The vector was kindly provided by Prof. J.-C. Perriard (Institute of Cell Biology, ETHZ, CH).

Motoneurons were isolated 74 h (stage 28 according to Hamburger and Hamilton [27]) after transfection as described by Kuhn [28]. Briefly, isolated spinal cord sections were collected in 4.5 ml PBS enriched with 5 g/l glucose and trypsinated by addition of 0.5 ml 2.5% Trypsin (2.5% 10×, Gibco). Enzyme treatment was stopped by addition of 0.5 ml horse serum (Gibco). Trypsin was removed by two centrifugation steps (14 min, 30 g, 20°C) followed by a resuspension of the cells in fresh culture medium. For aggregate formation, 2×10^6^ cells in 4 ml spinal cord cell culture medium were transferred into 25 ml Erlenmeyer flasks and incubated for 20 h on a gyratory shaker (HT, Infors AG, Bottmingen, Switzerland) at 81 rpm under cell culture conditions (37°C and 95% air/5% CO_2_). As cell culture medium we used Modified Minimum Essential Medium, supplemented with 2 mM glutamine, 5% heat-inactivated horse serum (all Invitrogen, CH), 2% chicken embryo extract, 1% nutrient mixture as described by Sonderegger et al [29] and 10% muscle cell conditioned medium (being the supernatant of embryonic chicken breast muscle cultures kept for 124 hours in this medium). Subsequently, cell reaggregates of optimal size (90–140 µm in diameter) were obtained. Thereafter, approximately 20 reaggregates in 5 µl medium were placed on the planar SiO_2_ surface of a chip near the topographic structures or on the teflon holder close to the fibers. The seeded reaggregates were incubated under cell culture conditions overnight to optimize adherence to the substrate. Seeded samples were later transferred into a sealed stainless steel chamber with a glass top cover to allow image acquisition. The chambers were perfused with oxygenated (95% air/5% CO_2_) cell culture medium, placed on a confocal laser scanning microscope stage (CLSM, Zeiss Axioplan 2 with LSM510 Scanning Module, 543/560 nm ex/em long pass filter) and heated to 37°C. Subsequently live monitoring of neurite outgrowth of motor neurons was performed [30]. Time lapse experiments were conducted for 16 h and digital images were acquired at 5 min intervals. We did not notice any laser induced effect on the viability or neurite outgrowth.

### Data collection and analysis

**Figure 3:**
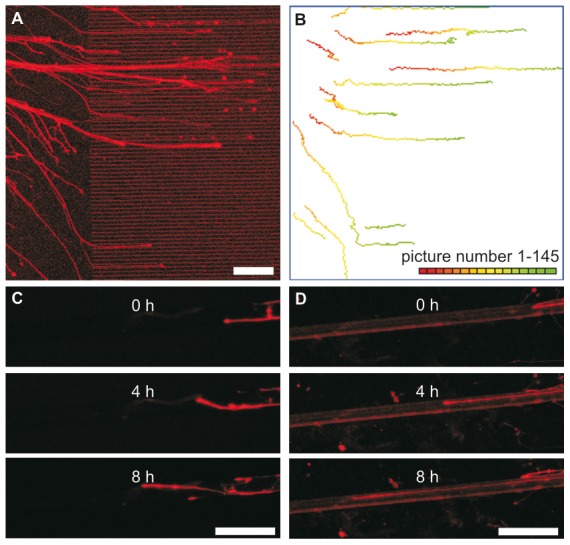
Fluorescent images and analysis of neurite outgrowth. A: Neurite outgrowth on a structured surface. The last picture taken of a live monitoring experiment (left) and the obtained trajectories (right) are shown. Neurites are growing from a single spinal cord reaggregate placed on the planar part into the ridge area (inter-ridge space 5 µm, ridge width 5 µm and ridge height 1.3 µm). B: Pictures of neurons growing on unstructured (left) or structured (right) PET fibers at different time points.

In order to describe and quantify growth cone behavior on the various surfaces, sequential fluorescence time lapse images were compared. The coordinates of selected growth cones were manually marked in each individual image [4]. Dislocation of the marked positions was analyzed using Visiometrics software [30], whereby the trajectories of single growth cones were generated in a semi-automated fashion (Fig. 3 B). Subsequently, growth cone behavior and migration was described in terms of velocity and direction as a function of each structure.

The following coefficients were defined:

v+: Mean growth cone forward migration velocity i.e. the mean dislocation of the growth cone per measurement interval (i.e. 5 min, from image to image) omitting retractions and zero velocities. For structured areas, the v+ value was calculated starting from the time lapse images following the first growth cone-ridge or growth cone-fiber contact.

The line perpendicular to the ridges was used as a reference line separating negative, zero and positive velocities. On unstructured surfaces, the line perpendicular to the line joining the first and the last growth cone locations was used as reference (Fig. S1).

v−: Mean retraction velocity. v− is identical to v+, but taking only retractions into account and excluding zero velocities.

In case of textile fibers as substrate, interpretation of growth perpendicular to fiber direction is very limited. Curvature distorts the determination of growth cone dislocation. For estimating forward and retracting velocity, only dislocations aligned along the fiber direction (0° or 180°±10°) expressed as v(0°) or v(180°) where taken into account

%v+: The frequency of time lapse points (at 5 min intervals) during which neurites protruded relative to the total number of periods in which the neurites retracted or protruded.

v_net_: Net growth cone dislocation per time unit (Δd in Fig. S1). Net growth is defined by the location of the growth cone at time t_r_ (at the first contact with the ridge or the fibers) and at time t_x_ (at the last measured position, see Fig. S1). v_net_ (expressed in µm/h) represents the mean effective (forward) velocity in the direction parallel to the ridges (x-axis), the main direction on a planar surface or the orientation of the fiber. In case of unstructured surfaces the starting point is the first picture at which the growth cone could be recognized. The v_net_ value is negatively affected by retractions and migration in the y-axis thus lower than v+.

% crossing: A ridge crossing is defined by the location of the growth cone at time t_r_ (at the first contact with the ridge) and at time t_x_ (at the last measured position, see Fig. S1). A crossing occurred if the value of Δr exceeds the width of a ridge. For the percentage of crossing, the number of crossing growth cones is divided by the total investigated neurites contacting the ridges.

α: The angle between the direction of the fiber and the vector defined by the location of the growth cone at time t = i and of time t = i+5 min. The direction of the fiber away from the reaggregates is defined as zero degree. The possible angles from 0 to 360 degrees are divided in classes with 10 degrees-steps. The frequency of occurring angles within each angle range was assessed.

The neurite growth on the different surfaces and fibers was analyzed till the observation period (16 h) or growth ended, as long as the growth cone was moving and as long as the growth cone was clearly distinguishable from other structures or cells. Only those neurons with growth cone trajectories of at least 160 min (planar surfaces) or 180 min (fibers) were included in the present study.

Statistical analysis of the data obtained from at least 3 independent experiments was performed using the ANOVA test with Bonferroni/Dunn multiple comparison analysis. In case of planar silicon surface the number of experiments was taken as independent and surface pattern type as dependent factor, whereas in case of textile fibers experiment fiber type and material were chosen as dependent and independent parameter, respectively. P-values less than 0.05 were defined as significant. All data are presented as mean ± SD.

## Results

In order to find best surface parameters for enhancing neurite outgrowth into a particular direction, we analyzed the displacement of growth cones using time lapse images (Fig. 3). In the first experimental series, we evaluated channel-like structures composed of SiO_2_ surface and PI ridges, with and without a-C:H coating. In the second series growth on textile surfaces was analyzed.

### Neurite outgrowth on structured planar surfaces

In general, we found spinal cord neurons to be unaffected by the a-C:H coating. They were morphologically indistinguishable (data not shown).

**Figure 4:**
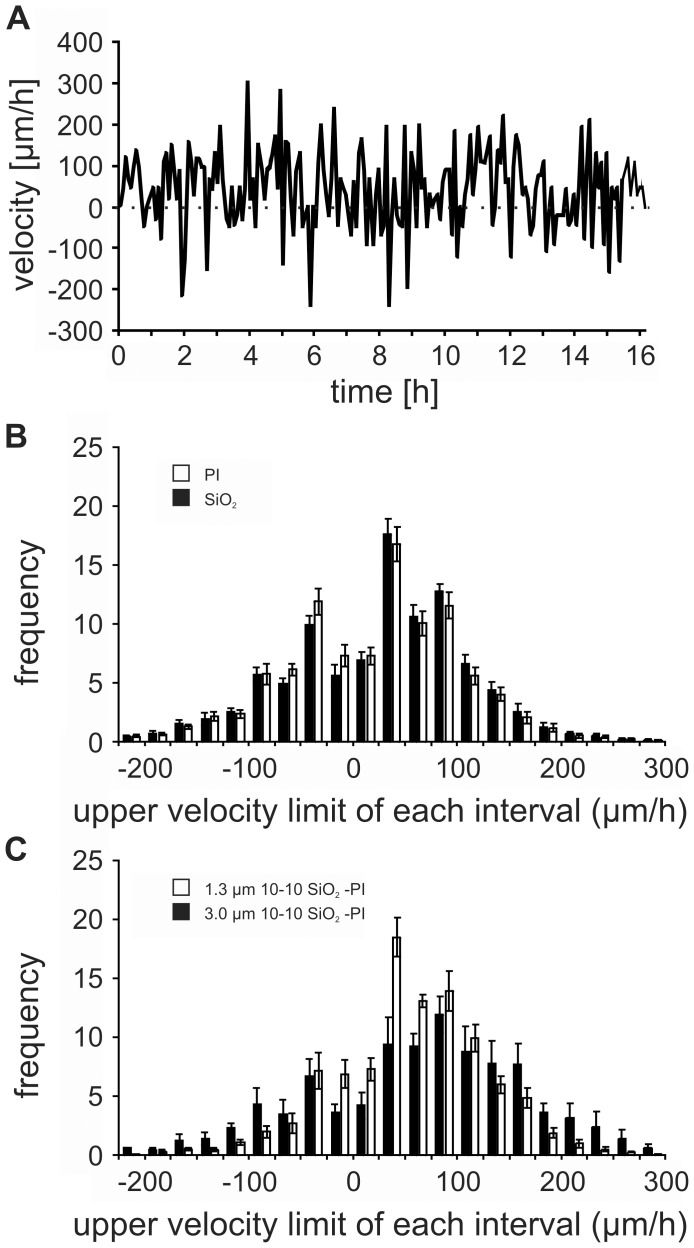
Neurite outgrowth on planar structured samples. A: Changes in velocity of an exemplary neurite. Neurites generally grow stepwise, with periods of relatively slow growth intercepted by bursts of rapid growth cone progression. B & C: Histogram of the frequency distribution of neurite outgrowth velocities on plane polyimide (PI) and SiO_2_ (B) and structured SiO_2_-PI surfaces with 10 µm wide ridges separated by an inter-ridge distance of 10 µm (C). The dislocation of the neurite growth cone after each 5 minutes period was taken to calculate the velocities. The velocities of all neurites measured on the mentioned surface were pooled. Each bar represents the velocity frequency over an interval of 25 µm/h. Below each bar only the upper velocity limit is given. Note that 4 peak frequencies were found corresponding to the velocity intervals: [(−100)–(−75)], [(−50)–(−25)], [25]–[50] and [75–100] (expressed in µm/h).

**Figure 5:**
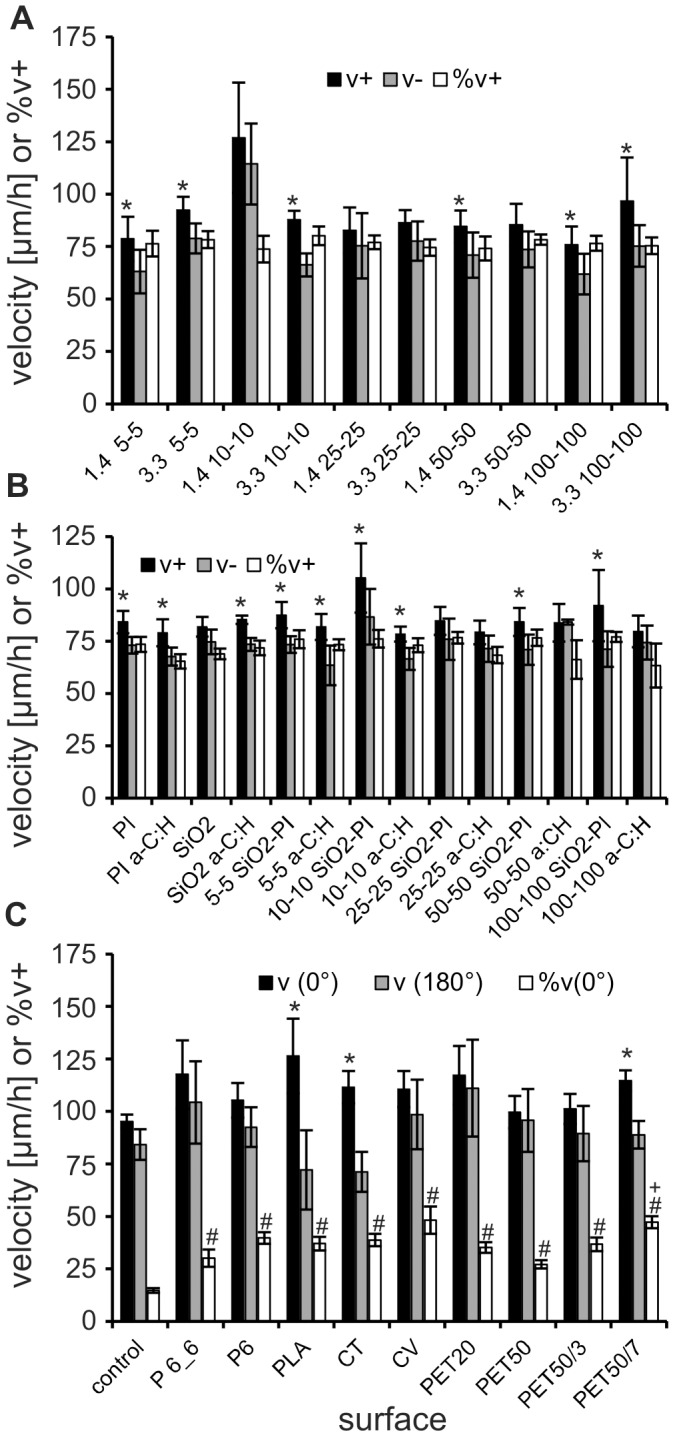
Comparison of growth cone outgrowth velocities (v+), retraction velocities (v−) velocities and the frequency of measured velocities being positive (%v+). Data are shown for experiments on SiO_2_-PI surfaces with ridges of different height and inter-ridge distances (A), on unstructured and structured surfaces with different inter-ridge distances (B, data of 1.3 and 3.0 µm height ridges pooled) and C on different structured or unstructured textile fibers and the respective control. Note: Due to the curvature of textile fibers only growth velocities along the axis of the fiber (0°±10°; 180°±10°) and the frequency of growth into fiber direction (%v(0°)) were analyzed in C. PI a-C:H and SiO_2_ a-C:H are planar polyimide and SiO_2_ surfaces coated with a-C:H. Asterisks (*; p<0.05) indicate significant different v+ compared to v− on the same surface, rhomb (#) significant different %v(0°) to control and a plus (+) significant different %v(0°) on fibers with grooves to PET50.

Neurite growth occurred stepwise with periods of relatively slow growth intercepted by bursts of rapid growth cone progression and retraction (Fig. 4 A). By separating the different periods of forward outgrowth and backward movement, we measured mean forward velocities (v+), backward velocities (v−), frequency of growth cone displacements in the forward direction (%v+) and net growth cone displacement (v_net_). We observed that these values were not significantly affected by a-C:H coating and surface structures (Fig. 4 B & C). The v+ values on the different surfaces were typically in the range of 80 to 90 µm/h whereas the v− values were significantly lower in most cases (about 18% less than v+, Fig. 5 A & B). We saw no significant difference in the velocities with respect to surface structure dimensions (ridge height, ridge width and spacing) and chemistry. Nonetheless, a tendency for increased migration values (v+ and v−) appeared to be associated with the inter-ridge distance of 10 µm compared to the other structural variables. v+ and v− values of over 100 µm/h have been found in this case. The frequency of growth cone displacements in the forward direction (%v+) on structured surfaces was in all cases between 70 and 80%, regardless of surface structure or chemistry (Fig. 5 A & B).

**Figure 6:**
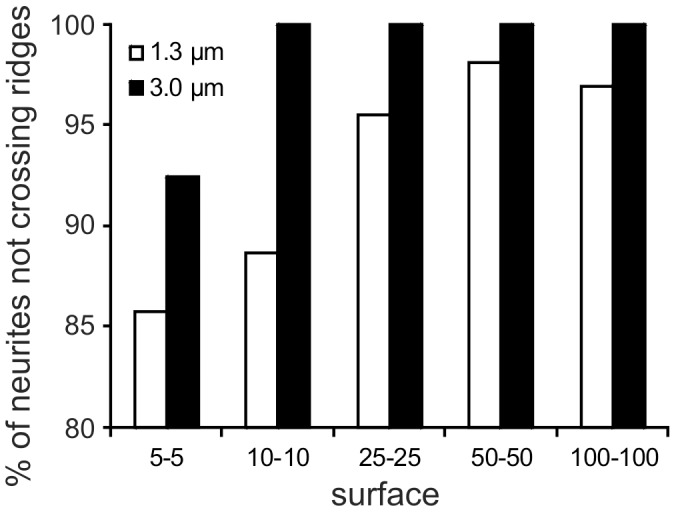
Percentage of neurites not crossing the ridges (data of all experiments using SiO_2_-PI and a-C:H surfaces were pooled). Values are based on behavior of 28 to 85 neurites. Note that for a ridge height of 3 µm none of the monitored growth cones crossed the ridges as far as the inter-ridge distance is 10 µm or more.

In addition to the growth velocity of the neurites, the exact guiding of growth into a specific direction is important. Thus, we analyzed what minimal height of the ridges and what channel width size is needed to keep the neurites inside the same “channel” and so how effectively they were guided by them. All neurites adjusted their growth trajectories when contacting ridge-structured areas (Fig. 3 A). Neurite orientation became parallel to the direction of the ridges and appeared to be confined to the channel-like structure between the ridges. As a result, neurite branching appeared to be strongly reduced, particularly where smaller inter-ridge spacing was present. Most growth cones stayed in contact with only one ridge. However, the tendency of the growth cones to cross the ridges increased when reducing the ridge height from 3.0 to 1.3 µm and also when decreasing the inter-ridge distance from 100 to 5 µm. For a ridge height of 3 µm none of the monitored growth cones crossed the ridges as long as the inter-ridge distance was 10 µm or more (Fig. 6).

**Figure 7:**
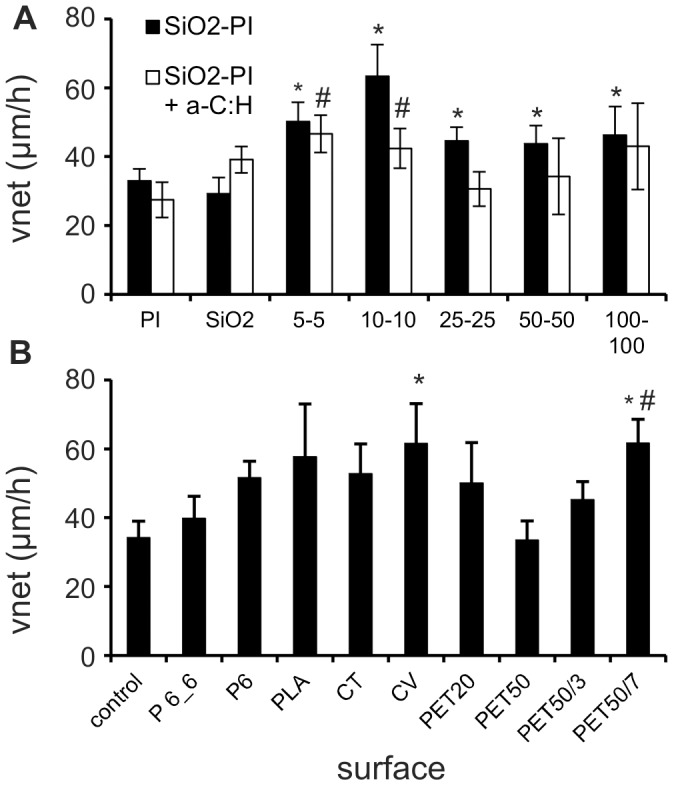
Net migration velocity. A: Net growth cone dislocation per time unit (v_net_) on planar PI or SiO_2_ surfaces and SiO_2_-PI surfaces with different ridge width and interridge distances, without or with a-C:H coating (data of 1.3 and 3.0 µm high ridges were pooled); *: p<0.05 significant different from uncoated SiO_2_; #: p<0.05 significant different from uncoated PI. B: Net outgrowth on the various fibers. *: p<0.05 significant different from control surface. #: p<0.05 significant different from PET50.

The v_net_ value was used as an indicator for the guiding effect, i.e. how far the structures are able to organize the back and forward movement into an effective forward movement. Here, growth speed and directionality of growth are important. The v_net_ values were approximately 50% of the corresponding values obtained for v+ and v− (Fig. 7). v_net_ was significantly higher on structured SiO_2_-PI surfaces in comparison to planar reference surfaces (SiO_2_ and PI), indicating a higher directionality of neurite growth by the ridges. For instance, growth cone v_net_ values on the surface area with 10-10 ridges were nearly twice the value of neurites grown on unstructured SiO_2_ and PI surfaces (Fig. 7).

### Neurite outgrowth on textile material

Stiff surfaces like SiO_2_ can hardly be implanted for supporting nerve regeneration. The best option would be a flexible material like textile fibers. As proof of concept, neurite growth was monitored on textile fibers of comparable diameters (16–21 µm) but different materials. As mentioned in the previous section, the migration of the growth cone showed phases of forward movement, absence of a clear dislocation and even backward movement (see also movie S1).

In contrast to the evaluated planar structured surfaces, textile fibers exhibit a curvature, which might distort the measured step size of growth cone dislocation. This impedes the calculation of v+, v− or %v+. Thus, we focused on the growth velocity and frequency of growth cone dislocations only in parallel forward (0°±10°; v(0°)) and backward (180°±10°; v(180°)) direction with respect to the fiber axis for investigating the guiding effect.

The frequency %v(0°) of growth steps in forward fiber direction ranged between 27.2% for PES 50 and 48.2% on viscose, which corresponds to a significant increase on all fibers compared to the control (14.7%; Fig. 5 C). However, the overall frequency %v+ of steps into forward direction on planar control surfaces (69.1%) was comparable to our results of the first experimental series on planar surfaces (70 to 80%).

On most textile fibers (without additional surface patterning by laser) v(0°) and v(180°) were slightly, but in no case significantly, higher than on the control surface (Fig. 5 C). The v(0°) ranged between 99.7 µm/h on control surfaces and 126.4 µm/h on PLA and was thus slightly higher than on planar surface (95.2 µm/h). The v(180°) were in most cases slightly lower and ranged between 71.2 µm/h (CT) and 76.8 µm/h (PET20) and also did not differ significantly from the control (84.2 µm/h). v(180°) was always slower than v(0°) (significant in the case of PLA and CT).

In order to analyze the effective guiding of growth cone movement, we calculated the velocity v_net_ (Fig. 7 B). Neurites that grew on planar control surfaces exhibited the lowest velocity (v_net_ = 34.2 µm/h) followed by the PA6.6 fibers (39.7 µm/h). PET20, PLA, PA6 and CT neurites showed similar v_net_ (50.0 to 61.6 µm/h). Net growth of neurites on CV was significantly faster (61.5 µm/h), whereas net growth on PES 50 fibers was in the similar range as the control (33.5 µm/h).

### Effects of fiber diameter

As a next step we investigated the possible impact of the fiber diameter on axonal growth. For this, neurite growth on PET fibers of 20 and 50 µm diameter was compared. Again, we did not detect significant differences in v(0°), v(180°), %v(0°) and v_net_ (Fig. 5 C, Fig. 7 B). However, neurites tend to grow slower on larger diameter PET fibers (v(0°): PET20 117.2 µm/h, PET50 99.7 µm/h and control 95.2 µm/h; v(180°): PET20 111.1 µm/h, PET50 95.7 µm/h and control 84.2 µm/h). The %v(0°) and v_net_ tended to be higher on fibers with smaller diameter (%v(0°) PET20 35.1% vs. PET50 27.1%; v_net_ PET20 50.0 µm/h vs. PET50 33.5 µm/h; Fig. 5 C & Fig. 7 B).

### Effects of fiber surface substructure

We have shown before, that longitudinal surface structures on planar surfaces are able to align neurites and to enhance the effective growth velocity. Therefore, as a final step we investigated how far guided growth on fibers can be improved by a grooved textile fiber surface. For this PET50 fibers with longitudinal grooves were fabricated (Fig. 2 C & F) using laser ablation.

Growth velocities (v(0°) & v (180°)) were again not affected by surface substructure (Fig. 5 C). The increase in effectiveness of guiding neurite growth by the grooves is evidenced by a groove depth dependent increase %v(0°). This increase was significant relative to fibers without groove in case of 7 µm deep grooves (Fig. 7 C; see also movies S1 & S2 in comparison). This resulted in a significant faster effective growth (v_net_) in 7 µm deep grooves (61.6 µm/h) and a tendency to faster growth in 3 µm deep grooves (45.1 µm/h; PET50: 33.5 µm/h).

## Discussion

The guiding of neurite growth for enhanced peripheral nerve regeneration and neuroimplants bridging critical size defects is a challenging topic of current neurobiology and medicine. In the present study we show that surface structuring of planar and even fiber surfaces are able to nearly double the net neurite outgrowth per time unit. The present finding may help to enlarge the size of the gap which can be bridged *in vivo* using conduits.

### Neurite outgrowth

The growth of neurites out of chicken embryonic spinal cord cell reaggregates were monitored by live imaging of fluorescent cells. Neurites behaved similar as the ones described in earlier studies [31]. Growth cones moved continuously but changed direction and velocity. Usually a major axonal orientation has been retained represented in around 70% movements in forward direction. But growth cones varied between forward movement, retraction and arresting around a position. Large differences (nearly 10 fold) have been reported for the neurite outgrowth velocities and all being lower than the velocity we measured here. For instance for chicken embryo DRG outgrowth velocities values between 4 and 63 µm/h on unstructured surfaces have been reported [32], [33], for rat DRG neurons 3 to 4 µm/h [34], for embryonic rat hippocampal neurons between 4 and 30 µm/h [35], [7], and for *Xenopus* spinal cord neurons between 14 and 70 µm/h [9], [36]. The mean velocities (v+, v−, v(0°), v(180°)) measured in the present study were around 80 µm/h and, thus, exceeding the range reported by others.

Theoretically, the difference in outgrowth velocities measured may be due to differences in nerve cell types, substratum and analyzing methods:

- Differences in nerve cell types. For different types of nerve cells similar broad velocity ranges were reported [7], [9], [32]–[36]. This suggests that the reported velocity differences are primarily not due to differences in the final nerve cell population that were investigated by the various research teams.
- Differences in substratum or cultures medium. Our results are consistent with previous studies, which show only relatively small effects of surface chemistry on neurite outgrowth [2], [37]. This suggests that the velocity differences can only partly be traced back on differences in coating procedure or presence or absence of growth factors.
- Differences in measurement methodology. Measuring the change in extent of nerve reaggregate or explant or the increase in neurite length after days or weeks does not allow to analyze growth dynamics [38], [39], [40] [7], [41], [42]. Analyzing neuronal length at certain time points repeatedly gives more accurate data on neurite outgrowth dynamics. Using time lapse microscopy, various recent studies also analyzed neurite outgrowth by sequentially comparing pictures of the same neurite at regular time intervals ranging from 3 min [43] to 60 minutes [2], [44], [45]. Apparently there is a dependency of growth velocity and length of measurement intervals. Lowest values (4 to 5 µm/h) are found in studies using endpoint assays after 24 or 48 h [34], [46], [47]. In time-lapse analysis with 6 h intervals an elongation rate of 16 µm/h for chicken DRG neurons was reported [48]. Higher frame rates of 1 h yielded values of about 60 µm/h were observed for *Xenopus* spinal cord [49] or chicken DRG neurons [50]. For rat hippocampal neurons the velocities measured were up to 20 µm/h [51], what is still fourfold higher than the values calculated after 24 h in an endpoint assay [47]. Higher temporal resolution (1 pict every 5 or 10 sec) revealed growth rates of up to 193.2 µm/h (mean 77.4 µm/h) for *Xenopus* neurons [52], which is in good agreement with the values gained in our study.

We observed that neurites generally grew stepwise, with periods of relatively slow growth intercepted by bursts of rapid growth cone progression or retraction. Studies with low temporal resolutions cannot notice those dynamics and are, therefore, misleading.

To show this, we calculated the net displacement (v_net_), which is comparable to an endpoint assay after 24 h to 48 h. We found that v_net_ values were around half of the values found for v+ and v−, thus, supporting our hypothesis.

The velocities found for outgrowth (v+) were around 18% higher than for retraction (v−). Growth cone translocation velocity is limited by the processes occurring within the growth cones. Anderson and co-workers [43] mentioned that advancing growth cones have more active filopodia than retracting rearward moving ones. Furthermore, the process of filopodia and lamellipodia protrusion and retraction depends among others on the rate of polymerisation and depolymerisation of cytoskeletal elements, like microtubules with a polymerisation rate exceeding the depolymerisation rate [53], [54]. Latter phenomena may explain the observed differences in v+ relative to v−.

### a-C:H and polyimide as potential biocompatible coatings

Various coating materials have been shown to have promising potential to be used for permanent implants. The potential of diamond-like carbon (DLC) coatings in influencing the growth of neurons was reported for embryonic chicken cerebral [55] and of rat cortex neurons [56]. The more elastic a-C:H possesses similar chemical and mechanical properties as DLC differing only in the presence of hydrogen in the carbon matrix. We have previously reported on the cytocompatibilty of a-C:H using rat bone marrow cells [57]. Accordingly, the compatibility of a-C:H with nerve cells was investigated in this study and is reported here, to the best of our knowledge, for the first time in literature. We could show here that the migration characteristics of nerve cells on a-C:H films (planar and structured) were similar to those on the reference SiO_2_ surfaces. SiO_2_ surfaces in combination with a poly-D-lysine surface treatment is known to support neurite outgrowth and to be biocompatible [58]. In addition, polyimide was evaluated. Polyimide has also been shown to be biologically inert and biocompatible [59]. It is an insulating material and is getting more and more attractive as flexible substrate for microelectronic, RFID applications and microfluidics (e.g. [60]). In this study, we could show that polyimide is not only cyto- but also biocompatible when taking neurite outgrowth into consideration. Neurites behaved in a similar way on planar polyimide and reference SiO_2_ surfaces. The appearance of the cultures on both surfaces was indistinguishable. This means that a-C:H and polyimide are interesting coatings for neuroimplants enabling to protect electronic and other sensitive parts for the aggressive biological environment.

### Neurite guidance and net neurite outgrowth on structured surfaces

For a support of neurite outgrowth by a material or conduit it is important that the neurite branching is reduced, the outgrowth is directed and the final location of single neurites can be defined. It has been shown in various studies that grooves and ridges are particular appropriate for steering neurite outgrowth and reducing branching [61], [62]. The groove and ridge dimensions play a crucial role in modulating neurite outgrowth. There is still a need, nonetheless, to define the precise dimensional characteristics necessary for the optimal control of growth guidance. As expected, the data obtained in this study strongly indicated that ridged surface structuring resulted in adjusting the trajectories of neurite growth almost upon contact. Spinal cord reaggregate neurites changed their orientation parallel to the ridges and became confined to the channel-like inter-ridge spaces. Additionally, the ridge-like structures reduced neurite branching in line with other studies [61], [63], [64]. We have demonstrated, however, that the degree of alignment is strongly linked to ridge dimensions, as 3 µm high ridges appeared to restrict neurite growth cone movement to single channels. This finding is in agreement with a study by Rajnicek et al. [9] showing that the percentage of hippocampal neurites crossing the ridges was dramatically reduced by increasing ridge height. According to their data about 70% of the neurites crossed ridges with a height of 1.3 µm and only 35% ridges with a height of 3.0 µm, with the remaining neurites growing in parallel to the grooves [9]. A similar behavior has been observed for embryonic chick cerebral neurons in case of steps [8]. The phenomenon was even more pronounced on micromachined grooved surfaces on which 1 µm deep grooves were without effect, whereas 2 µm deep grooves provided strong alignment [65]. In line with brain derived neurons, the alignment of neonatal rat DRG in 10 µm wide and 3 µm deep grooves was increased from 76 to 92% by increasing the depth to 4 µm [37]. In the study presented here, we could show for the first time that spinal cord neurons not only behave in a similar way but that the impact of the topographic structure is even more pronounced.

In addition to the role of ridge height, ridge width and inter-ridge spacing are equally important in modulating neurite growth. In this respect, ridge height and inter-ridge distance appear to synergistically affect spinal cord neurite outgrowth. The presence of such synergism is described by Rajnicek et al. [9], demonstrating that the growth orientation of embryonic rat hippocampal neurites changed from perpendicular to parallel to the grooves by increasing the groove depth (up to 1.1 µm) and width (up to 4 µm) [9]. We have shown in this study that the frequency of the neurites to cross the ridges was not only reduced by increasing the ridge height from 1.3 to 3.0 µm, but also by increasing the inter-ridge distance (Fig. 6). Summarizing our findings, we conclude that a ridge height of 3.0 µm and an inter-ridge distance of 10 µm may present an ideal combination for the design interfaces where the guidance and modulation of single neurites is crucial.

Beside guidance of neurites for improving the accuracy of nerve regeneration [66], also the net growth cone displacement is of key importance. By measuring v_net_ instead of v+ or v− on SiO_2_-PI surfaces, 10–10 µm ridges were able to promote the outgrowth significantly (by a factor of two) in analogy to the findings of [9] and [62]. Furthermore, *in vivo* maximal neurite net displacement of conditioned regenerating peripheral nerves between two fluorine resin films was found to be 418 µm/day, corresponding to 17 µm/h [67]. In the present study, on planar surfaces a comparable net displacement was found on PI surfaces (25 µm/h). Thus, the height of the ridges, inter-ridge distance as well as surface chemistry seem not or only negligibly affect the outcome and the functionality of the nerve cells but are able to significantly promote effective (net) neurite outgrowth.

### Textile fibers as a possible nerve guiding substrate

For supporting neuronal regeneration in the body, flexible materials are to be preferred. Recently, various teams were able to guide neuron outgrowth successfully by applying aligned fibers of different materials as surfaces [20], [21], [22], [23] or by sponges with uniaxial pores building channels through the scaffold with pore diameters of 20 to 50 µm [68] and 120 µm [69]. However, only Lietz et al [70] optimized fibers surfaces with artificial microstructured to additionally enhance neurite growth. They found triangular microgrooves of about 2–3 µm opening width and 6 µm depth to induce straight-lined neurite growth in contrast to a trend for meandering growth on unstructured filaments.

In our time-lapse series we found neurites growing more straight-line on fibers than on planar surfaces. This is presumably affected by the curvature and natural microstructures on the surface. The bending stiffness of the cytoskeleton may limit growth on substrates with high curvature [71] and, thus, induce growth along the axis of fibers. A slight increase in v_net_ and %v(0°) in smaller diameter supports this hypothesis.

Longitudinal topography, either natural (CV) or artificial, enhanced the amount of growth cone dislocation into direction of fiber axis significantly. Grooves width a depth of 7 µm enhanced neuron guidance successfully. Grooves of lower depth indeed tended to guide neurite growth, but values were not significant. Our structures show a V-shaped with an opening of at least twice the groove opening (i.e. 2–3 µm) employed by [70]. The slope of the channel walls may play here a crucial role. Presumably, by increasing the depths the shape get less important with the deeper the channels the more efficient the neurite outgrowth guidance is.

## Conclusions

We conclude that the neurite growth analyzing methods used here are adequate for detailed analysis of neurite growth on different materials. a-C:H and polyimide are biocompatible coating materials. Furthermore, topographic structures in the low micrometer range have a strong influence on the outgrowth behavior of spinal cord neurons and may therefore be used to optimize surfaces for directed neurite growth. Highest guiding efficiency was achieved by topographical cues of 3 µm in height and 10 µm inter-ridge distance. Moreover, these cues do not alter nerve functionality taking outgrowth and retraction velocity as quality index, but are able to promote net growth cone displacement. The transfer of our findings on textile fibers demonstrated a similar growth promoting effect, which might lead to adapted nerve scaffolds with higher regeneration velocity and possibly increase of nerve gap size that can be bridged.

## Supporting Information

Figure S1 **Schematic representation of the migration behavior of two neurites (a) and (b).** Neurite (a) does not and (b) does contact the ridges during the time frame of the experiment. In case of neurite (a) the vector Δd is defined by the first and last picture of the experiment. In case of neurite (b) the vector Δd is defined by the moment of contact and the last picture. Δr defines in how far the neurite crossed the ridge(s). A neurite is considered to cross the ridge if Δr exceeds the inter-ridge distance.
(DOCX)

Movie S1 **Time lapse series of an exemplary experiment with RFP-transfected neurites growing on an unstructured PET50 fiber.** The movie is showing 12 h of growth with a picture taken every 5 min.
(MOV)

Movie S2 **Time lapse series of an exemplary experiment with RFP-transfected neurites growing on a structured PET50/7 fiber.** The movie is showing 12 h of growth with a picture taken every 5 min.
(MOV)

## References

[1] Ming GL, Wong ST, Henley J, Yuan XB, Song HJ, et al (2002) Adaptation in the chemotactic guidance of nerve growth cones. Nature 417: 411–418. 11986620 10.1038/nature745

[2] Song HJ, Ming GL, Poo MM (1997) cAMP-induced switching in turning direction of nerve growth cones. Nature 388: 276–279. 10.1038/40864 9230436

[3] Cao X, Shoichet MS (2001) Defining the concentration gradient of nerve growth factor for guided neurite outgrowth. Neurosci 103: 831–840. 10.1016/s0306-4522(01)00029-x 11274797

[4] Adams DN, Kao EYC, Hypolite CL, Distefano MD, Hu WS, et al (2005) Growth cones turn and migrate up an immobolized gradient of the laminin IKVAV peptide. J Neurobiol 62: 134–147. 15452851 10.1002/neu.20075

[5] Torimitsu K, Kawana A (1990) Selective growth of sensory nerve fibers on metal oxide pattern in culture. Devl Brain Res 51: 128–131. 10.1016/0165-3806(90)90265-z 2297889

[6] Bernard A, Fitzli D, Sonderegger P, Delamarche E, Michel B, et al (2001) Affinity capture of proteins from solution and their dissociation by contact printing. Nat Biotechnol 19: 866–869. 11533647 10.1038/nbt0901-866

[7] Dowell-Mesfin NM, Abdul-Karim MA, Turner AMP, Schanz S, Craighead HG, et al (2004) Topgraphically modified surfaces affect orientation and growth of hippocampal neurons. J Neural Eng 1: 78–90. 15876626 10.1088/1741-2560/1/2/003

[8] Clark P, Connolly P, Curtis AS, Dow JA, Wilkinson CD (1987) Topographical control of cell behaviour: I. Simple step cues. Development 99: 439–448. 3653011 10.1242/dev.99.3.439

[9] Rajnicek AM, Britland S, McCaig CD (1997) Contact guidance of CNS neurites on grooved quartz: influence of groove dimensions, neuronal age and cell type. J Cell Sci 110: 2905–2913. 9359873 10.1242/jcs.110.23.2905

[10] Stier H, Schlosshauer B (1995) Axonal guidance in the chicken retina. Development 121: 1443–1454. 7789274 10.1242/dev.121.5.1443

[11] Sun M, Kingham PJ, Reid AJ, Armstrong SJ, Terenghi G, et al (2010) In vitro and in vivo testing of novel ultrathin PCL and PCL/PLA blend films as peripheral nerve conduit. J Biomed Mater Res A 93: 1470–1481. 19967758 10.1002/jbm.a.32681

[12] Morelli S, Salerno S, Piscioneri A, Papenburg BJ, Di Vito A, et al (2010) Influence of micro-patterned PLLA membranes on outgrowth and orientation of hippocampal neurites. Biomaterials 31: 7000–7011. 20579728 10.1016/j.biomaterials.2010.05.079

[13] Reich U, Fadeeva E, Warnecke A, Paasche G, Muller P, et al (2012) Directing neuronal cell growth on implant material surfaces by microstructuring. J Biomed Mater Res B Appl Biomater 100: 940–947. 22287482 10.1002/jbm.b.32656

[14] Mahoney MJ, Chen RR, Tan J, Saltzman WM (2005) The influence of microchannels on neurite growth and architecture. Biomaterials 26: 771–778. 15350782 10.1016/j.biomaterials.2004.03.015

[15] Wieringa PA, Wiertz RW, de Weerd E, Rutten WL (2010) Bifurcating microchannels as a scaffold to induce separation of regenerating neurites. J Neural Eng 7: 16001. 20054102 10.1088/1741-2560/7/1/016001

[16] Clark P, Connolly P, Curtis ASG, Dow JAT, Wilkinson CDW (1991) Cell guidance by ultrafine topography in vitro. J Cell Sci 99: 73–77. 1757503 10.1242/jcs.99.1.73

[17] Kaehr B, Allen R, KJavier DJ, Currie J, Shear JB (2004) Guiding neuronal development with in situ microfabrication. PNAS 101: 16104–16108. 15534228 10.1073/pnas.0407204101 PMC528953

[18] Nagata I, Kawana A, Nakatsuji N (1993) Perpendicular contact guidance of CNS neuroblasts on artificial microstructures. Development 117: 401–408. 8223260 10.1242/dev.117.1.401

[19] Yucel D, Kose GT, Hasirci V (2010) Polyester based nerve guidance conduit design. Biomaterials 31: 1596–1603. 19932504 10.1016/j.biomaterials.2009.11.013

[20] Klinkhammer K, Bockelmann J, Simitzis C, Brook GA, Grafahrend D, et al (2010) Functionalization of electrospun fibers of poly(epsilon-caprolactone) with star shaped NCO-poly(ethylene glycol)-stat-poly(propylene glycol) for neuronal cell guidance. J Mater Sci Mater Med 21: 2637–2651. 20567886 10.1007/s10856-010-4112-7

[21] Schnell E, Klinkhammer K, Balzer S, Brook G, Klee D, et al (2007) Guidance of glial cell migration and axonal growth on electrospun nanofibers of poly-epsilon-caprolactone and a collagen/poly-epsilon-caprolactone blend. Biomaterials 28: 3012–3025. 17408736 10.1016/j.biomaterials.2007.03.009

[22] Kim YT, Haftel VK, Kumar S, Bellamkonda RV (2008) The role of aligned polymer fiber-based constructs in the bridging of long peripheral nerve gaps. Biomaterials 29: 3117–3127. 18448163 10.1016/j.biomaterials.2008.03.042 PMC2483242

[23] Madduri S, Papaloizos M, Gander B (2010) Trophically and topographically functionalized silk fibroin nerve conduits for guided peripheral nerve regeneration. Biomaterials 31: 2323–2334. 20004018 10.1016/j.biomaterials.2009.11.073

[24] Francz G, Schroeder A, Hauert R (1999) Surface analysis and bioreactions to Ti and V conatining a-C:H. Surf Interface Anal 28: 3–7.

[25] Wagner F, Hoffmann P (2000) The angle dependence of structure formation on excimer laser ablated ramps in stretched poly(ethylene terephthalate). Applied Surface Science 168: 158–161.

[26] Pekarik V, Bourikas D, Miglino N, Joset P, Preiswerk S, et al (2003) Screening for gene function in chicken embryo using RNAi and electroporation. Nat Biotechnol 21: 93–96. 12496763 10.1038/nbt770

[27] Hamburger V, Hamilton HL (1951) A Series of Normal Stages in the Development of the Chick Embryo. Journal of Morphology 88: 49–&. 1304821 10.1002/aja.1001950404

[28] Kuhn TB (2003) Growing and working with spinal motor neurons. Meth Cell Biol 71: 67–87. 10.1016/s0091-679x(03)01005-7 12884687

[29] Sonderegger P, Lemkin PF, Lipkin LE, Nelson PG (1985) Differential modulation of the expression of axonal proteins by non-neuronal cells of the peripheral and central nervous system. EMBO J 4: 1395–1401. 4029116 10.1002/j.1460-2075.1985.tb03792.x PMC554357

[30] Kaiser JP, Reinmann A, Bruinink A (2006) The effect of topographic characteristics on cell migration velocity. Biomaterials 27: 5230–5241. 16814858 10.1016/j.biomaterials.2006.06.002

[31] Katz MJ (1985) How Straight Do Axons Grow. Journal of Neuroscience 5: 589–595. 3973686 10.1523/JNEUROSCI.05-03-00589.1985 PMC6565018

[32] Stepien E, Stanisz J, Korohoda W (1999) Contact guidance of chick embryo neurons on single scratches in glass and on underlying aligned human skin fibroblasts. Cell Biol Int 23: 105–116. 10561119 10.1006/cbir.1998.0256

[33] Schlosshauer B, Müller E, Schröder B, Planck H, Müller HW (2003) Rat schwann cells in bioresorbabble nerve guides to promote and accelerate axonal regeneration. Brain Res 963: 321–326. 12560139 10.1016/s0006-8993(02)03930-6

[34] Miller C, Jeftinija S, Mallapragada S (2002) Synergistic effects of physical and chemical guidance cues on neurite alignment and outgrowth on biodegradable polymer substrates. Tissue Engineering 8: 367–378. 12167224 10.1089/107632702760184646

[35] Rajnicek AM, McCaig CD (1997) Guidance of CNS growth cones by substratum grooves and ridges: effects of inhibitors of the cytoskeleton, calcium channels and signal transduction pathways. J Cell Sci 110: 2915–2824. 9359874 10.1242/jcs.110.23.2915

[36] Rajnicek AM, Foubister LE, McCaig CD (2006) Temporally and spatially coordinated roles for Rho, Rac, Cdc42 and their effectors in growth cone guidance by a physiological electric field. J Cell Sci 119: 1723–1735. 16595546 10.1242/jcs.02896

[37] Miller C, Jeftinija S, Mallapragada S (2002) Synergistic effects of physical and chemical guidance cues on neurite alignment and outgrowth on biodegradable polymer substrates. Tissue Eng 8: 367–378. 12167224 10.1089/107632702760184646

[38] Shah AK, Fischer C, Knapp CF, Sisken BF (2004) Quantitation of neurite growth parameters in explant cultures using a new image processing program. J Neurosci Meth 136: 123–131. 10.1016/j.jneumeth.2004.01.010 15183264

[39] Bilsland J, Rigby M, Young L, Harper S (1999) A rapid method for semi-quantitative analysis of neurite outgrowth from chick DRG explants using image analysis. J Neurosci Meth 92: 75–85. 10.1016/s0165-0270(99)00099-0 10595705

[40] Tai H, Buettner HM (1998) Neurite outgrowth and growth cone morphology on micropatterned surfaces. Biotechnol Prog 14: 364–370. 9622516 10.1021/bp980035r

[41] Bouquet C, Soares S, von Boxberg Y, Ravaille-Veron M, Propst F, et al (2004) Microtubule-associated protein 1B controls directionality of growth cone migration and axonal branching in regeneration of adult dorsal root ganglia neurons. J Neurosci 24: 7204–7213. 15306655 10.1523/JNEUROSCI.2254-04.2004 PMC6729172

[42] Brown MD, Cornejo BJ, Kuhn TB, Bamberg JR (2000) Cdc42 stimulates neurite outgrowth and formation of growth cone filopodia and lamellipodia. J Neurobiol 43: 352–364. 10861561 10.1002/1097-4695(20000615)43:4<352::aid-neu4>3.0.co;2-t

[43] Anderson M, Boström M, Pfaller K, Glueckert R, Schrott-Fischer A, et al (2006) Structure and locomotion of adult in vitro regenerated spiral ganglian growth cones - A study using video microscopy and SEM. Hearing Res 215: 97–107. 10.1016/j.heares.2006.03.014 16684592

[44] Dotti CG, Sullivan CA, Banker GA (1988) Te establishment of polarity by hippocampal neurons in culture. J Neurosci 8: 1454–1468. 3282038 10.1523/JNEUROSCI.08-04-01454.1988 PMC6569279

[45] Odde DJ, Tanaka EM, Hawkins SS, Buettner HM (1996) Stochastic dynamics of the nerve growth cone and its microtubules during neurite outgrowth. Biotech Bioeng 50: 452–461. 10.1002/(SICI)1097-0290(19960520)50:4<452::AID-BIT13>3.0.CO;2-L 18626995

[46] Stepien E, Stanisz J, Korohoda W (1999) Contact guidance of chick embryo neurons on single scratches in glass and on underlying aligned human skin fibroblasts. Cell Biology International 23: 105–116. 10561119 10.1006/cbir.1998.0256

[47] Rajnicek AM, Britland S, McCaig CD (1997) Contact guidance of CNS neurites on grooved quartz: influence of groove dimensions, neuronal age and cell type. Journal of Cell Science 110: 2905–2913. 9359873 10.1242/jcs.110.23.2905

[48] Song M, Uhrich KE (2007) Optimal micropattern dimension enhance neurite outgrowth rates, lengths, and orientations. Annals of Biomedical Engineering 10.1007/s10439-007-9348-0 17616821

[49] Rajnicek AM, Foubister LE, McCaig CD (2006) Temporally and spatially coordinated roles for Rho, Rac, Cdc42 and their effectors in growth cone guidance by a physiological electric field. Journal of Cell Science 119: 1723–1735. 16595546 10.1242/jcs.02896

[50] Schlosshauer B, Muller E, Schroder B, Planck H, Muller HW (2003) Rat Schwann cells in bioresorbable nerve guides to promote and accelerate axonal regeneration. Brain Research 963: 321–326. 12560139 10.1016/s0006-8993(02)03930-6

[51] Dotti CG, Sullivan CA, Banker GA (1988) The Establishment of Polarity by Hippocampal-Neurons in Culture. Journal of Neuroscience 8: 1454–1468. 3282038 10.1523/JNEUROSCI.08-04-01454.1988 PMC6569279

[52] Odde DJ, Tanaka EM, Hawkins SS, Buettner HM (1996) Stochastic dynamics of the nerve growth cone and its microtubules during neurite outgrowth. Biotechnology and Bioengineering 50: 452–461. 18626995 10.1002/(SICI)1097-0290(19960520)50:4<452::AID-BIT13>3.0.CO;2-L

[53] Dent EW, Callaway JL, Szebenyi G, Baas PW, Kalil K (1999) Reorganization and movement of microtubules in axonal growth cones and developing interstitial branches. J Neurosci 19: 8894–8908. 10516309 10.1523/JNEUROSCI.19-20-08894.1999 PMC6782770

[54] Tanaka EM, Kirschner MW (1991) Microtubule behaviour in the growth cones of living neurons during axon elongation. J Cell Biol 115: 345–363. 1918145 10.1083/jcb.115.2.345 PMC2289161

[55] Ignatius MJ, Sawhney N, Gupta A, Thibadeau BM, Monteiro OR, et al (1998) Bioactive surface coatings for nanoscale instruments: Effects on CNS neurons. J Biomed Mat Res 40: 264–274. 10.1002/(sici)1097-4636(199805)40:2<264::aid-jbm11>3.0.co;2-m 9549621

[56] Kelly S, Regan EM, Uney JB, Dick AD, McGeehan JP, et al (2008) Patterned growth of neuronal cells on modified diamond-like carbon substrates. Biomaterials 29: 2573–2580. 18359076 10.1016/j.biomaterials.2008.03.001

[57] Schroeder A, Francz G, Bruinink A, Hauert R, Mayer J, et al (2000) Titanium containing amorphous hydrogenated carbon films (a-C: H/Ti): surface analysis and evaluation of cellular reactions using bone marrow cell cultures in vitro. Biomaterials 21: 449–456. 10674809 10.1016/s0142-9612(99)00135-0

[58] Corey JM, Brunette AL, Chen MS, Weyhenmeyer JA, Brewer GJ, et al (1997) Differentiated B104 neuroblastoma cells are a high-resolution assay for micropatterned substrates. J Neurosci Meth 75: 91–97. 10.1016/s0165-0270(97)00062-9 9262149

[59] Kim Y-H, Hwang E-S, Kim Y-J (2005) Polymer/metal based flexible MEMS biosensors for nerve signal monitoring and sensitive skin. J Semiconductor Technol Sci 5: 11–16.

[60] Lee TH, Pan H, Kim IS, Kim JK, Cho TH, et al (2010) Functional Regeneration of a Severed Peripheral Nerve With a 7-mm Gap in Rats Through the Use of An Implantable Electrical Stimulator and a Conduit Electrode With Collagen Coating. Neuromodulation 13: 299–305. 21992887 10.1111/j.1525-1403.2010.00296.x

[61] Mahoney MJ, Chen RR, Tan J, Saltzman WM (2005) The influence of microchannels on neurite growth and architecture. Biomaterials 26: 771–778. 15350782 10.1016/j.biomaterials.2004.03.015

[62] Morelli S, Salerno S, Piscioneri A, Papenburg BJ, Di Vito A, et al (2010) Influence of micro-patterned PLLA membranes on outgrowth and orientation of hippocampal neurites. Biomaterials 31: 7000–7011. 20579728 10.1016/j.biomaterials.2010.05.079

[63] Hirono T, Torimitsu K, Kawana A, Fukuda J (1988) Recognition of artificial microstructures by sensory nerve fibers in culture. Brain Res 446: 189–194. 3370482 10.1016/0006-8993(88)91314-5

[64] Yao L, Wang S, Cui W, Sherlock R, O'Connell C, et al (2009) Effect of functionalized micropatterned PLGA on guided neurite growth. Acta Biomat 5: 580–588. 10.1016/j.actbio.2008.09.002 18835227

[65] Clark P, Conolly P, Curtis A (1990) Topographical control of cell behaviour: II. Multiple grooved substrata. Development 108: 635–644. 2387239 10.1242/dev.108.4.635

[66] Trumble TE, Archibald S, Allan CH (2004) Bioengineering for nerve repair in the future. J Am Soc Surg Hand 4: 134–142.

[67] Torigoe K, Tanaka H, Takahashi A, Awaya A, Hashimoto K (1996) Basic behavior of migratory Schwann cells in peripheral nerve regeneration. Exp Neruol 137: 301–308. 10.1006/exnr.1996.0030 8635545

[68] Bozkurt A, Deumens R, Beckmann C, Olde Damink L, Schügner F, et al (2009) In vitro cell alignment obtained with a Schwann cell enriched microstructured nerve guide with longitudinal guidance channels. Biomaterials 30: 169–179. 18922575 10.1016/j.biomaterials.2008.09.017

[69] Zhang Q, Zhao Y, Yan S, Yang Y, Zhao H, et al (2012) Preparation of uniaxial multichannel silk fibroin scaffolds for guiding primary neurons. Acta Biomaterialia 8: 11. 10.1016/j.actbio.2012.03.033 22465574

[70] Lietz M, Dreesmann L, Hoss M, Oberhoffner S, Schlosshauer B (2006) Neuro tissue engineering of glial nerve guides and the impact of different cell types. Biomaterials 27: 1425–1436. 16169587 10.1016/j.biomaterials.2005.08.007

[71] Smeal RM, Rabbitt R, Biran R, Tresco PA (2005) Substrate curvature influences the direction of nerve outgrowth. Annals of Biomedical Engineering 33: 376–382. 15868728 10.1007/s10439-005-1740-z

