# Supplementary material for: Surface Microstructures on Planar Substrates and Textile Fibers Guide Neurite Outgrowth: A Scaffold Solution to Push Limits of Critical Nerve Defect Regeneration?
Source: PLoS One. 2012 Dec 12;7(12):e50714. doi: 10.1371/journal.pone.0050714 (PMC3520951; doi:10.1371/journal.pone.0050714)

**Supplementary data**

**Fig S1**: Schematic representation of the migration behavior of two neurites (a) and (b). Neurite (a) does not and (b) does contact the ridges during the time frame of the experiment. In case of neurite (a) the vector Δd is defined by the first and last picture of the experiment. In case of neurite (b) the vector Δd is defined by the moment of contact and the last picture. Δr defines in how far the neurite crossed the ridge(s). A neurite is considered to cross the ridge if Δr exceeds the inter-ridge distance.


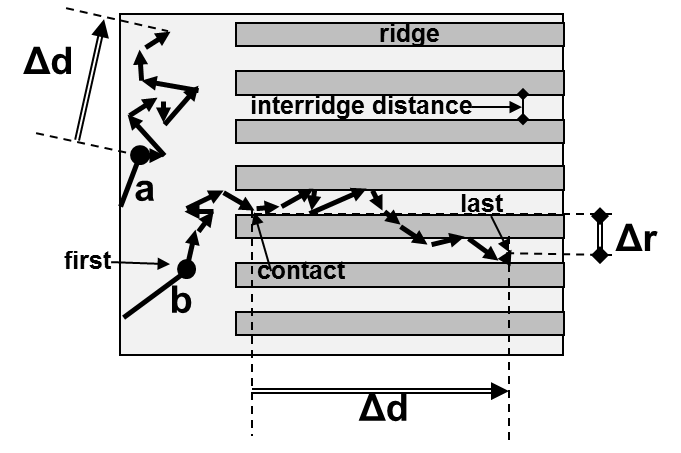

Supplement: Figure S1 — Schematic representation of the migration behavior of two neurites (a) and (b). Neurite (a) does not and (b) does contact the ridges during the time frame of the experiment. In case of neurite (a) the vector Δd is defined by the first and last picture of the experiment. In case of neurite (b) the vector Δd is defined by the moment of contact and the last picture. Δr defines in how far the neurite crossed the ridge(s). A neurite is considered to cross the ridge if Δr exceeds the inter-ridge distance. (DOCX) [file pone.0050714.s001.docx]
